# Long Non-Coding RNAs: Significant Drivers of Carcinogenesis Mechanisms in Head and Neck Squamous Cell Carcinoma

**DOI:** 10.3390/cimb47090698

**Published:** 2025-08-28

**Authors:** Camelia Mia Hotnog, Marinela Bostan, Matei Anghelescu, Viviana Roman, Coralia Bleotu, Razvan Hainarosie, Catalina Voiosu, Stefania Marineata, Ioana-Stefania Bostan, Carmen Cristina Diaconu, Mirela Mihaila

**Affiliations:** 1 Center of Immunology, Stefan S. Nicolau Institute of Virology, Romanian Academy, 030304 Bucharest, Romania; camelia.hotnog@virology.ro (C.M.H.); mirela.mihaila@virology.ro (M.M.); 2 Department of Biochemistry and Biophysics, Faculty of Midwives and Nursing, University of Medicine and Pharmacy Carol Davila, 050474 Bucharest, Romania; 3 Department of Immunology, ‘Victor Babes’ National Institute of Pathology, 050096 Bucharest, Romania; 4 Independent Researcher, 013156 Bucharest, Romania; mateianghelescu1@gmail.com; 5 Department of Cellular and Molecular Pathology, Stefan S. Nicolau Institute of Virology, Romanian Academy, 030304 Bucharest, Romania; coralia.bleotu@virology.ro (C.B.); carmen.diaconu@virology.ro (C.C.D.); 6 Research Institute of the University of Bucharest (ICUB), University of Bucharest, 060023 Bucharest, Romania; 7 Otorhinolaryngology and Head and Neck Surgery Department, Faculty of Medicine, University of Medicine and Pharmacy Carol Davila Bucharest, 050474 Bucharest, Romania; razvan.hainarosie@umfcd.ro (R.H.); catalina.pietrosanu@umfcd.ro (C.V.); stefania.marineata@umfcd.ro (S.M.); 8 Virology Department, Faculty of Medicine, University of Medicine and Pharmacy Carol Davila Bucharest, 050474 Bucharest, Romania; 9 Institute of Phonoaudiology and Functional ENT Surgery, 061344 Bucharest, Romania; 10 Filantropia Clinical Hospital, 011132 Bucharest, Romania; ioana-stefania.bostan@rez.umfcd.ro; 11 Faculty of Pharmacy, Titu Maiorescu University, 040314 Bucharest, Romania

**Keywords:** lncRNA, HNSCC, carcinogenesis, gene regulation, metastasis, epigenetics, therapy

## Abstract

Head and neck squamous cell carcinoma (HNSCC) is an aggressive cancer with a complex molecular landscape. Despite extensive research, our understanding of the molecular mechanisms remains incomplete, hindering the development of effective therapeutic strategies for this disease. Long non-coding RNAs (lncRNAs) have emerged as crucial factors in cancer biology, regulating key networks across various malignancies. These molecules exert their regulatory functions through interactions with nucleic acids or proteins, thereby influencing signaling pathways within tumor cells. Consequently, lncRNAs play a significant role in key processes like cell proliferation, metastasis, immune evasion, and treatment resistance. This review offers a comprehensive overview of current knowledge regarding lncRNA-mediated mechanisms in HNSCC. The first section explores how lncRNAs influence tumor processes through various modulation mechanisms, including transcriptional and post-transcriptional regulation, chromatin remodeling, and epigenetic modifications. We also highlight the impact of lncRNAs on specific signaling pathways that control essential cellular functions (e.g., proliferation, apoptosis, angiogenesis, invasion, metastasis). Ultimately, this underscores the promising potential of lncRNAs as diagnostic biomarkers and therapeutic targets capable of enhancing patient care in oncology. Gaining a deep understanding of how lncRNAs modulate carcinogenic mechanisms may yield innovative approaches for early detection, personalized treatment, and improved clinical outcomes for HNSCC patients.

## 1. Introduction

Head and neck squamous cell carcinoma (HNSCC) encompasses a group of biologically diverse malignancies arising from the mucosal epithelium of the oral cavity, pharynx, and larynx. This type of cancer is associated with risk factors like tobacco and alcohol use, as well as human papillomavirus (HPV) infections. Globally, HNSCC accounts for over 800,000 new cases and more than 430,000 deaths annually, making it the sixth most common cancer worldwide [1]. Many patients are diagnosed at advanced stages, frequently involving metastasis to cervical lymph nodes [2]. Despite advances in treatment strategies that often include combined therapies, incorporating surgery, radiotherapy, chemotherapy, and immunotherapy, the prognosis for individuals with HNSCC remains notably poor, highlighting the need for continuous research and innovation [3,4]. In the last years, molecular research has emerged as a critical avenue for improving diagnostic accuracy, prognostic stratification, and therapeutic efficacy in HNSCC. Understanding the molecular underpinnings of tumorigenesis is essential for the development of targeted interventions and personalized treatment approaches.

Some transcriptomic studies have identified a variety of long non-coding RNAs (lncRNAs) that may play crucial roles in biological processes relevant to this type of cancer [5,6]. lncRNAs are RNA molecules greater than 200 nucleotides in length that do not encode proteins, and they have emerged as key regulatory elements. They have complex regulatory functions, capable of interacting with DNA, RNA and proteins, thereby modulating gene expression at transcriptional, post-transcriptional, and epigenetic levels [7], thus influencing epigenetics, transcription, and transcript stability. Through this versatility, they can act as “central nodes” in tumor signaling networks. In the context of HNSCC, lncRNAs deserve special attention due to the multiple roles they fulfill in tumor biology. Functionally, lncRNAs contain specific regions for protein binding or sequences that can associate with microRNAs (miRNAs), frequently enriched in repeated RNA motifs that impart structural or regulatory functions [8].

Unlike miRNAs (which have broad targets) or coding genes (which give “driver” mutations), lncRNAs offer greater tissue/subtype specificity, directly reflect epigenetic dysregulation, are stable in liquid biopsies, and can be both biomarkers and therapeutic targets. lncRNAs directly reflect epigenetic dysregulation and can be measured non-invasively (plasma/saliva). For example, HOTAIR and MALAT1 are detectable in blood and elevated in patients with poor prognosis, while LINC00460 differentiates HPV+ from HPV− tumors and thus may be useful for patient stratification.

Within the nucleus, these lncRNAs can influence chromatin states and regulate transcription, while in the cytoplasm they may affect mRNA stability, translation, and signaling pathways.

Emerging evidence suggests that lncRNA expression is a significant driver of tumor development, progression, and therapeutic resistance in HNSCC [9,10]. This review aims to explore the role of lncRNAs in the pathogenesis of HNSCC by highlighting their functions and mechanisms of action. By elucidating their roles in tumor biology, we highlight the promising potential of lncRNAs as diagnostic and prognostic tools and their viability as therapeutic targets. While recognizing the challenges encountered in future research efforts, this information offers new insights for improving clinical outcomes in patients with HNSCC.

## 2. Mechanisms of lncRNA-Mediated Carcinogenesis in HNSCC

**Figure 1:**
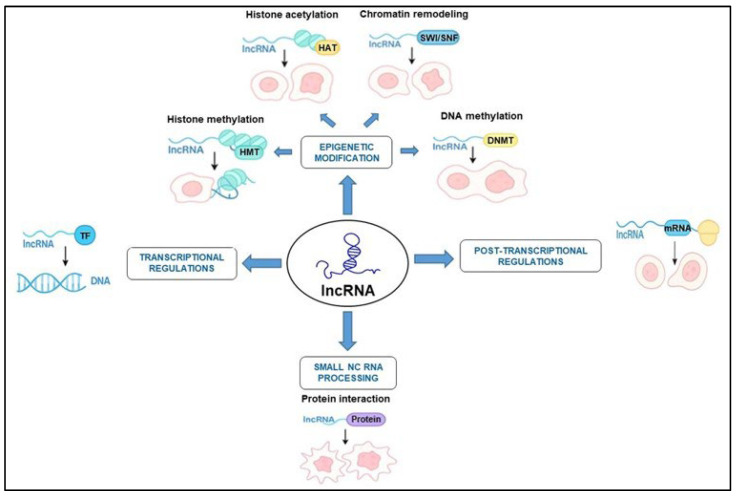
Role of lncRNAs in the development of HNSCC by affecting epigenetic changes, transcription, and post-transcriptional processes. These complex mechanisms contribute to oncogene activation, repression of tumor suppressor genes, and resistance to therapies.

lncRNAs contribute to the development and progression of cancer through a variety of molecular mechanisms, functioning at multiple regulatory levels. In HNSCC, lncRNAs exert critical roles in transcriptional and post-transcriptional regulation. These regulatory mechanisms enable lncRNAs to modulate gene expression, cellular behavior, and tumor progression by interacting with transcriptional machinery, mRNA molecules, and RNA-binding proteins (Figure 1).

### 2.1. Epigenetic Regulation of HNSCC by lncRNAs

In HNSCC, lncRNAs are increasingly recognized as key epigenetic regulators, contributing to carcinogenesis. Through interactions with chromatin-modifying complexes, lncRNAs contribute to tumorigenesis by altering gene expression without modifying the DNA sequence. They influence histone modification, DNA methylation, and chromatin architecture, thereby promoting oncogenic transformation. Many lncRNAs act as molecular scaffolds or guides that recruit chromatin-modifying enzymes to specific gene loci, leading to epigenetic silencing of tumor suppressors or activation of oncogenes. HOX transcript antisense RNA (HOTAIR) is one of the most well-studied lncRNAs with epigenetic function. In HNSCC, it interacts with Polycomb Repressive Complex 2 (PRC2), particularly EZH2, to induce trimethylation of histone H3 at lysine 27 (H3K27me3), a repressive mark, leading to chromatin compaction and gene silencing—especially of tumor suppressor genes, such as PTEN and E-cadherin, thereby promoting proliferation, invasion, and metastasis [11]. Another lncRNA, LINC00460, plays an important role in the DNA methylation and silencing of tumor suppressor genes processes. In oral squamous cell carcinoma (OSCC), a subtype of HNSCC, LINC00460 is overexpressed and recruits DNA methyltransferase 1 (DNMT1) to the miR-612 promoter, promoting DNA methylation and silencing of this tumor-suppressive microRNA. This leads to upregulation of downstream oncogenes and enhanced epithelial–mesenchymal transition (EMT) [12,13].

By guiding epigenetic modifiers to tumor suppressor loci, lncRNAs can silence critical genes involved in cell cycle arrest (e.g., CDKN1A/p21) or apoptosis (modulation of p53, PI3K/AKT). This repression facilitates uncontrolled proliferation and genomic instability. Metastasis-associated lung adenocarcinoma transcript 1 (MALAT1) is an lncRNA that modulates gene expression by affecting the distribution of active histone marks and regulating alternative splicing through interaction with splicing factors. In HNSCC, MALAT1 is linked to metastasis and poor prognosis [14].

Some lncRNAs can recruit epigenetic activators to enhance oncogene expression. Plasmacytoma Variant Translocation 1 (PVT1), which is frequently upregulated in HNSCC, can interact with chromatin remodelers to enhance the transcription of MYC, a key oncogene, by stabilizing chromatin loops or modulating enhancer activity [15]. Some lncRNAs interact with ATP-Dependent Chromatin Remodeling Complexes, such as SWI/SNF complexes (e.g., BRG1-containing complexes). These interactions help reposition nucleosomes, ultimately leading to the activation or repression of gene expression [16,17]. While the role of these mechanisms in HNSCC is not yet fully understood, ongoing research suggests that lncRNAs may play a significant role in promoting cancer stemness and therapy resistance [18].

The epigenetic regulation mediated by lncRNAs in HNSCC plays a critical role in tumor initiation and progression (Figure 1). These lncRNAs represent attractive targets for epigenetic therapy, particularly by disrupting their interactions with chromatin-modifying enzymes like EZH2 or DNMT1. Aberrant expression of chromatin-modulating lncRNAs correlates with poor prognosis, lymph node metastasis, and resistance to radiotherapy in HNSCC patients. The expression of epigenetically active lncRNAs can aid in early detection or prognosis.

### 2.2. Role of lncRNAs in Transcriptional and Post-Transcriptional Regulation in HNSCC

In HNSCC, lncRNAs exert critical roles in transcriptional and post-transcriptional regulation. These regulatory mechanisms enable lncRNAs to modulate gene expression, cellular behavior, and tumor progression by interacting with transcriptional machinery, mRNA molecules, and RNA-binding proteins [19,20] (Figure 1).

#### 2.2.1. Transcriptional Regulation

lncRNAs play a crucial role in regulating transcription by interacting with DNA, transcription factors, and chromatin regulators. They can either activate or repress gene expression. lncRNAs can interact with transcription factors and act as decoys or co-activators, modulating the availability or activity of transcription factors (Figure 1). In HNSCC, LINC00673 enhances the transcription of Slug (a key EMT transcription factor) by interacting with EZH2, repressing E-cadherin, and promoting invasion and metastasis [21]. Some lncRNAs function similarly to enhancers, promoting the transcription of nearby or distant genes by altering chromatin structure or looping. An example is CCAT1, which regulates MYC expression through long-range chromatin interactions, contributing to proliferation in OSCC [22]. Additionally, lncRNAs can guide repressor complexes to the promoters of tumor suppressor genes, leading to a reduction in their transcription.

Other lncRNAs may also act as transcriptional regulators involved in tumor progression in HNSCC, such as MALAT1, which modulates alternative splicing and regulates gene expression through nuclear interactions, being responsible for amplifying tumor proliferation and invasion; PVT1, which transcriptionally regulates genes involved in cell proliferation and survival; and H19, which acts as a “sponge” for microRNAs and influences the expression of some oncogenes at the transcriptional level [23].

#### 2.2.2. Post-Transcriptional Control

Post-transcriptional regulation encompasses the stability and distribution of various transcripts, including mechanisms like alternative splicing, nuclear degradation (via the exosome), processing, and nuclear export, in which RNA-binding proteins (RBPs) frequently assume a critical role. This regulation may also extend to protein modifications and the subcellular localization of proteins (Figure 1). Disruptions in these processes have been linked to tumorigenesis [24]. In the context of HNSCC, specific lncRNAs can significantly influence mRNA splicing, stability, and translation. These lncRNAs may bind to RBPs or directly to mRNAs, thereby modulating their degradation or translation efficiency. This modulation subsequently alters the levels of pivotal oncogenic or tumor-suppressive proteins [25]. Moreover, lncRNAs may function as molecular scaffolds, facilitating the recruitment of RBPs to specific pre-mRNAs and modifying isoform expression profiles that favor cancer progression. For instance, MALAT1 is an lncRNA that modulates alternative splicing by engaging with serine/arginine-rich splicing factors (SRSFs) and additionally stabilizes mRNAs associated with epithelial–mesenchymal transition (EMT) and angiogenesis [26]. HOTAIR is similarly recognized for its ability to bind RBPs, including heterogeneous nuclear ribonucleoproteins (hnRNPs), thereby influencing splicing and stability across various cancers, including HNSCC [27].

There is an increasing body of evidence indicating that other lncRNAs contribute to the stabilization or degradation of target mRNAs. TINCR is an example of an lncRNA that stabilizes differentiation-associated mRNAs by forming lncRNA–mRNA duplexes in HNSCC [28]. Furthermore, lncRNAs can interact with RBPs, such as HuR (ELAVL1), IGF2BP family proteins, or AUF1, thereby either stabilizing or destabilizing target mRNAs. In HNSCC, elevated expression of certain lncRNAs can enhance the stability of oncogenic mRNAs by serving as decoys or by participating in the formation of stabilizing RBP–mRNA complexes [29].

miRNA sponging (Competing Endogenous RNA, ceRNA) is one of the most common post-transcriptional roles of lncRNAs in sequestering tumor-suppressive miRNAs, preventing them from degrading their target mRNAs (usually oncogenes). Some studies mention that interactions between non-coding RNAs (lncRNAs) and microRNAs may occur in RNA regulatory processes [30,31]. The “sponge” mechanism functions by preventing microRNAs from repressing their target messenger RNAs. As a result, this mechanism leads to the upregulation of oncogenes and the downregulation of tumor suppressor genes, thereby contributing to the process of carcinogenesis in HNSCC.

## 3. Impact of lncRNAs on the Modulation of Signaling Pathways That Govern Cellular Functions in HNSCC

**Figure 2:**
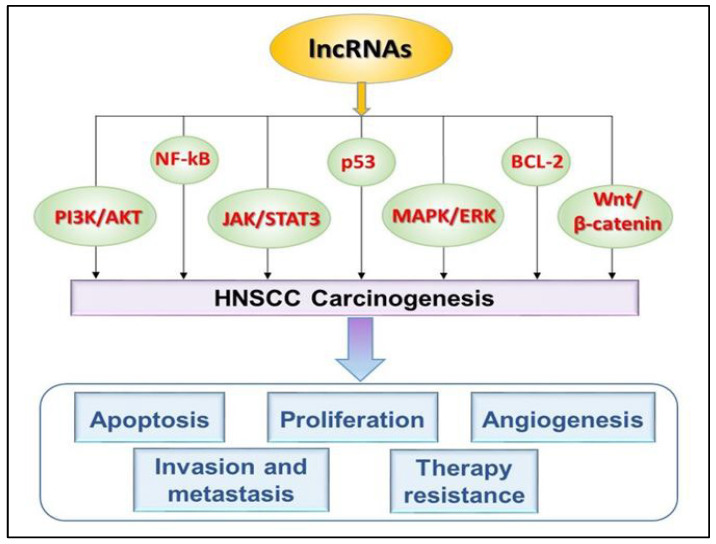
Role of lncRNAs in the modulation of signaling pathways that govern cellular functions in HNSCC. lncRNAs can modulate the most important signaling pathways in the cell (PI3K/AKT, JAK/STAT3, Wnt/β-catenin, MAPK/ERK, etc.), having the ability to regulate all of the processes involved in HNSCC carcinogenesis (apoptosis, cell proliferation, angiogenesis, invasion, metastasis, and resistance to therapy).

lncRNAs play a critical role in the modulation of key oncogenic pathways through several distinct mechanisms. These include acting as scaffolds to facilitate the assembly of proteins, serving as decoys to sequester proteins or microRNAs (miRNAs), functioning as guides to recruit chromatin-modifying proteins, operating as sponges that bind miRNAs in a manner akin to competitive endogenous RNAs (ceRNAs), and enhancing transcriptional processes. Interactions between lncRNAs and signaling pathways can result in a variety of biological effects, such as the promotion of cellular proliferation, activation of invasion and metastasis, induction of stemness qualities, modulation of immune responses (including the expression of immune checkpoints), and the development of resistance to therapeutic interventions, such as chemotherapy and radiotherapy [32] (Figure 2).

### 3.1. lncRNAs’ Modulation of Signaling Pathways Involved in HNSCC Apoptosis

Apoptosis is the process of programmed cell death, which is crucial for removing damaged or unwanted cells. Cancer cells evade apoptosis to survive and proliferate. In cancer contexts (including HNSCC and other solid tumors), lncRNAs frequently regulate signaling pathways that control apoptosis, either suppressing it (most common in oncogenic lncRNAs) or promoting it (for tumor-suppressive lncRNAs). Oncogenic lncRNAs generally suppress apoptosis by activating survival pathways (AKT, NF-κB, STAT3), silencing tumor suppressors (p53, PTEN), upregulating anti-apoptotic proteins (BCL-2), and inhibiting death receptor pathways. Tumor-suppressor lncRNAs (e.g., MEG3) can enhance apoptosis by activating p53 or suppressing survival signaling [33] (Table 1, Figure 2).

The PI3K/AKT/mTOR pathway represents a critical survival signaling cascade in HNSCC. This pathway is frequently dysregulated in HNSCC due to various factors, including genetic mutations, amplifications, such as PIK3CA, loss of the tumor suppressor PTEN, and overexpression of EGFR. Thus, it plays a significant role in inhibiting apoptosis and promoting cellular survival. Certain lncRNAs contribute to the prevention of apoptosis by sustaining AKT activity. For example, HOTAIR functions to suppress apoptosis by silencing PTEN, thereby increasing AKT activation [34,35]. Similarly, UCA1 (urothelial cancer-associated 1) serves as a sponge for miR-193a, which leads to an increase in PI3K activity and enhanced phosphorylation of AKT. Additionally, MALAT1 can sponge miR-200c, induce decreased PTEN, and indirectly activate AKT signaling. Hyperactivation of the PI3K/AKT/mTOR pathway is frequently observed in HNSCC. This hyperactivation contributes to resistance to apoptosis and therapeutic interventions, such as cisplatin and radiotherapy (Figure 2). lncRNAs modulate this pathway through diverse mechanisms, rendering them potential biomarkers for both diagnosis and prognosis, as well as promising therapeutic targets to overcome drug resistance and improve patient outcomes. As a result, targeting this pathway with PI3K inhibitors, AKT inhibitors, and mTOR inhibitors represents an active area of clinical research to restore the sensitivity of tumors to apoptosis [36].

The p53 pathway—the “guardian of the genome”—is the key tumor suppressor that induces apoptosis in response to DNA damage and oncogenic stress. It acts to activate the transcription of pro-apoptotic genes (e.g., BAX, PUMA, NOXA) or to suppress anti-apoptotic genes (e.g., BCL-2). p53 mutations are highly frequent in HNSCC (~50–70% of cases, especially in HPV-negative tumors). Loss of p53 function leads to evasion of apoptosis, resulting in increased cell survival and accumulation of mutations, which contributes to tumor progression [37]. In HNSCC, p53 pathway dysfunction is a critical driver of apoptosis resistance and poor therapeutic outcomes (Figure 2). Oncogenic lncRNAs suppress p53-mediated apoptosis, while tumor-suppressive lncRNAs enhance it. lncRNAs, such as HOTAIR and MALAT1, inhibit p53-mediated apoptosis through epigenetic silencing and miRNA sponging, contributing to tumor progression and treatment resistance (Table 1). Conversely, tumor-suppressor lncRNAs like MEG3 can activate p53 signaling to promote apoptosis. Targeting these lncRNAs represents a promising strategy to restore p53 function and improve clinical outcomes in HNSCC patients [38].

BCL-2 family regulation is crucial in balancing pro-apoptotic proteins (such as BAX and BAK) and anti-apoptotic proteins (like BCL-2 and BCL-XL). The BCL-2 family controls mitochondrial (intrinsic) apoptosis. Overexpression of anti-apoptotic BCL-2 family members is common in HNSCC. Tumor cells upregulate BCL-2, BCL-XL, and MCL-1, allowing them to evade apoptosis even in the presence of DNA damage or therapy. This mechanism is associated with resistance to chemotherapy agents, such as cisplatin and 5-fluorouracil (5-FU), and radiotherapy. Furthermore, it may correlate with poor prognosis and aggressive clinical behavior [39]. Specific lncRNAs upregulate the anti-apoptotic protein BCL-2, thus blocking mitochondrial apoptosis. In HNSCC, dysregulation of BCL-2 family proteins, often through overexpression of anti-apoptotic members, contributes to apoptosis resistance and treatment failure. lncRNAs, such as HOTAIR and MALAT1, upregulate BCL-2 via epigenetic and ceRNA mechanisms, promoting tumor survival. Conversely, tumor-suppressor lncRNAs like MEG3 and GAS5 can enhance pro-apoptotic signaling [40,41]. Targeting these lncRNAs represents a promising strategy to modulate BCL-2 family dynamics, overcome therapeutic resistance, and improve clinical outcomes. lncRNAs’ signatures can assist in stratifying patients based on predicted therapy responses [42]. Upregulated lncRNAs enhance the expression of anti-apoptotic BCL-2 family members, contributing to resistance against chemotherapy and radiotherapy (Figure 2). Clinical relevance is highlighted by strategies aimed at blocking lncRNAs, which could make tumors more sensitive to apoptosis-inducing therapies. Techniques like antisense oligonucleotides (ASOs), small interfering RNAs (siRNAs), and CRISPR methods are employed to silence oncogenic lncRNAs. Additionally, using BCL-2 family inhibitors, such as venetoclax, can help restore the apoptosis process. These approaches offer potential for personalized medicine based on lncRNA-BCL-2 profiles [43,44].

NF-κB (Nuclear Factor kappa-light-chain-enhancer of activated B cells) signaling contributes to cell survival, inflammation, and the expression of anti-apoptotic genes. Activation of the NF-κB pathway involves the following steps: phosphorylation of IκB leads to its degradation, allowing NF-κB to translocate to the nucleus and initiate gene transcription [45]. NF-κB is constitutively active in many HNSCC tumors, primarily driven by chronic inflammation (e.g., smoking, alcohol), viral oncogenes (e.g., HPV E6/E7), or genetic alterations (e.g., IκBα mutations, TRAF amplification). lncRNAs sustain NF-κB activity and suppress apoptosis. For example, MALAT1 activates NF-κB, leading to the upregulation of anti-apoptotic genes, such as BCL-XL and XIAP [46]. Similarly, HNF1A-AS1 promotes the degradation of IκB by regulating E3 ligases or kinases (such as the IKK complex) that phosphorylate IκB, thereby promoting NF-κB activation [47]. HOTAIR recruits chromatin modifiers to enhance NF-κB target gene expression, promoting anti-apoptotic and pro-survival effects [48] (Table 1, Figure 2).

The JAK/STAT3 (Janus Kinase/Signal Transducer and Activator of Transcription 3) pathway is involved in cytokine-driven survival signaling, where STAT3 induces the expression of anti-apoptotic genes. MALAT1 and LINC00152 enhance STAT3 phosphorylation and sustain the upregulation of anti-apoptotic genes [49] (Table 1).

The MAPK/ERK (Mitogen-Activated Protein Kinase/Extracellular Signal-Regulated Kinase) pathway is a major signaling cascade that activates the expression of anti-apoptotic genes (BCL-2, MCL-1) and inhibits pro-apoptotic proteins (BIM, BAD) via phosphorylation. In HNSCC, the MAPK/ERK pathway is frequently hyperactivated, promoting anti-apoptotic signaling and therapy resistance (Table 1). Long non-coding RNAs, such as HOTAIR, MALAT1, and UCA1, enhance ERK signaling via epigenetic regulation and ceRNA mechanisms, leading to increased expression of anti-apoptotic genes like BCL-2 [50]. Also, AFAP1-AS1 promotes ERK phosphorylation, contributing to anti-apoptotic signaling. Conversely, tumor-suppressor lncRNAs, such as GAS5 and MEG3, can inhibit ERK activity, restoring apoptotic sensitivity [51,52]. Overexpression of ERK-activating lncRNAs (HOTAIR, MALAT1) correlates with poor prognosis and is associated with aggressive disease and lymph node metastasis. Hyperactive MAPK/ERK signaling leads to resistance to apoptosis, which results in the failure of treatments like cisplatin, 5-FU, and radiation therapy [53] (Figure 2). lncRNAs driving this hyperactivation are implicated in primary and acquired resistance. Targeting these lncRNAs or combining lncRNA-targeted therapy with MEK/ERK inhibitors represents a promising strategy to modulate ERK-driven apoptosis resistance and improve clinical outcomes in HNSCC. Personalized medicine approaches assure the selection of patients for ERK inhibitor therapy based on lncRNA expression [54,55].

The death receptor pathways, including FAS, TNF, and TRAIL, serve as extrinsic apoptosis triggers via receptor–ligand interactions. In cancer, including HNSCC, tumor cells often suppress death receptor signaling, leading to apoptosis evasion and resistance to chemotherapy and radiotherapy (Figure 2). Tumors can downregulate receptors, increase decoy receptors, or upregulate inhibitors like c-FLIP [56]. Certain lncRNAs suppress extrinsic apoptosis pathways. HOTAIR has been reported to indirectly reduce the expression of death receptors or their ligands through mechanisms like miRNA sponging or chromatin remodeling [57]. MALAT1 suppresses death receptor signaling by sponging miRNAs that typically enhance death receptor expression or by increasing inhibitors like c-FLIP, which blocks Caspase-8 activation [58]. GAS5 is generally pro-apoptotic, but its downregulation in HNSCC may lead to a reduction in apoptosis mediated by death receptors [59] (Table 1).

### 3.2. lncRNAs’ Modulation of Signaling Pathways Involved in HNSCC Proliferation

Uncontrolled cellular division, characterized by cell proliferation, is a fundamental hallmark of cancer. Tumor cells divide uncontrollably because they lose normal cell cycle checkpoints. Cell proliferation is dependent on cell cycle progression, particularly transitions, such as from the G1 to the S phase. This progression is regulated by cyclins (e.g., cyclin D1 and cyclin E) and cyclin-dependent kinases (CDKs) (e.g., CDK4 and CDK6), along with their inhibitors (e.g., p21 and p27). The overexpression of cyclins and CDKs drives uncontrolled cancer growth [60]. In HNSCC, lncRNAs have become key regulators in tumor growth and progression. lncRNAs promote the proliferation of HNSCC cells by modulating the cell cycle by upregulating cyclins and CDKs, suppressing CDK inhibitors, sponging tumor-suppressive miRNAs, activating oncogenic signaling pathways (PI3K/AKT/mTOR, MAPK/ERK), and recruiting epigenetic modifiers to silence cell cycle checkpoints [61].

Some lncRNAs act in the nucleus to recruit transcription factors or co-activators to cyclin gene promoters. They can increase the transcription or stability of cyclin D1, cyclin E, and CDK4/6, thereby facilitating the G1/S transition and accelerating cell division. For example, MALAT1 upregulates cyclin D1 via PI3K/AKT signaling (Table 2). UCA1 activates AKT, and these pathways increase cyclin expression (cyclin D1) and reduce CDK inhibitors (p21/p27) [62]. HOTAIR is among the most extensively characterized lncRNAs in HNSCC, and it enhances Wnt/β-catenin signaling by epigenetically silencing Wnt inhibitors. This process promotes the nuclear translocation of β-catenin, resulting in increased binding of β-catenin to the cyclin D1 promoter and subsequent transcription activation [63] (Table 2). Additionally, lncRNAs act as ceRNAs that “sponge” the miRNAs, thereby preventing these miRNAs from inhibiting the expression of cyclins and CDKs. For example, H19 functions as a ceRNA that sponges miR-675, suppressing Wnt pathway components (Table 2). This activation of the Wnt/β-catenin signaling cascade leads to an accumulation of β-catenin in the nucleus, further increasing its interaction with the cyclin D1 promoter and driving the transition from the G1 to the S phase of the cell cycle [64]. Similarly, MALAT1 sponges miR-101 and promotes the expression of CDK1 [65].

Moreover, HOTAIR can contribute to epigenetic remodeling by recruiting chromatin modifiers (e.g., EZH2/PRC2) to activate cyclin gene promoters; it can reduce repressive histone marks, opening chromatin for transcription. HOTAIR guides PRC2 to the promoters of tumor suppressor genes, such as CDK inhibitors, like p21 and p16. PRC2 methylates histone H3 at lysine 27 (H3K27me3), a repressive chromatin mark. This silences the transcription of CDK inhibitors, leading to decreased levels of p21 and p16 and diminishing the inhibition of CDKs, thereby accelerating cell cycle progression and contributing to the aggressive growth of tumors. Furthermore, HOTAIR exerts a simultaneous effect by activating oncogenes, such as cyclin D1, while repressing tumor suppressors like p21. This dual epigenetic modulation is a hallmark of its oncogenic role in HNSCC [11]. Overexpression of HOTAIR in HNSCC is correlated with elevated levels of cyclin D1, suggesting a relationship with aggressive cell proliferation and a negative prognosis [66]. Thus, in HNSCC, lncRNAs promote cell proliferation by activating major oncogenic pathways, such as EGFR/PI3K/AKT/mTOR, MAPK/ERK, Wnt/β-catenin, JAK/STAT3, and MYC (Figure 2). They act via miRNA sponging, chromatin remodeling, and mRNA stabilization, highlighting their role as key drivers of tumor growth and potential therapeutic targets.

### 3.3. Impact of lncRNAs on the Signaling Pathways Involved in HNSCC Angiogenesis

Angiogenesis is a crucial step in cancer progression, taking into account that neovascularization is indispensable to the formation of solid tumors, as the formation of new blood vessels facilitates the tumor’s access to oxygen and nutrients. In addition, the increased permeability of newly formed blood vessels is favorable for the metastasis of tumor cells. Tumor angiogenesis is regulated by a complex interaction of pro-angiogenic and anti-angiogenic factors. Among the many factors that mediate angiogenesis, VEGF (vascular endothelial growth factor) is known as the key regulator at the initial stage of tumor angiogenesis. Angiopoietin 2 (Ang2) plays a particular role in vessel maturation, while MMP9 is an important pro-angiogenic factor that induces ECM degradation and has been proven to be regulated by multiple signaling pathways, such as the PI3K/AKT, ERK, and JAK/STAT pathways [67,68].

Recently, several lncRNAs have been studied and shown to be important regulators of angiogenic factors. For example, an anti-angiogenetic effect of HOTAIR knockdown was observed in nasopharyngeal cancer (NPC), both in vitro and in vivo, by directly suppressing the expression and secretion of VEGF-A and Ang2 in NPC cells and animal xenograft, providing evidence that HOTAIR silencing acts as an anti-angiogenesis factor in NPC carcinogenesis [69]. Similarly, knockdown of FOXCUT (FOXC1 Upstream Transcript) lncRNA led to the downregulation of angiogenesis in OSCC-derived Tca8113 and SCC9 cell lines. After FOXCUT and FOXC1 (an EMT-related gene) silencing, the expression levels of MMP2, MMP7, MMP9, and VEGF-A were downregulated, suggesting a possible role for the FOXC1/FOXCUT gene pair in tumor angiogenesis in OSCC [70]. LINC00319 is an lncRNA that can be modulated by chemokines, being a downstream molecule of Chemokine ligand 18 (CCL18), which is involved in tumor microenvironment regulation and plays a critical role in several cancers, including OSCC. It has been found that LINC00319 knockdown attenuates the carcinogenic function of CCL18 in OSCC, reducing proliferation, metastasis, epithelial–mesenchymal transition, and angiogenesis. Furthermore, overexpression of LINC00319 increased the levels of VEGFA and MMP9, promoting the angiogenic ability of OSCC cells [71] (Table 3).

Another mechanism for lncRNAs to mediate tumor angiogenesis involves carcinoma-associated fibroblasts (CAFs). CAFs, often known as activated fibroblasts, play a central role in the tumor microenvironment and can promote tumor angiogenesis by secreting higher levels of proteolytic enzymes, such as matrix metalloproteinases [72]. In a recent study, Xu et al. showed the regulatory role of FENDRR (FOXF1 adjacent non-coding developmental regulatory RNA) on the pro-angiogenic effect of CAFs derived from OSCC patients. FENDRR is an lncRNA shown to be expressed at low levels in a variety of tumor tissues and to inhibit tumor progression [73]. Also, in OSCC patients, FENDRR has a downregulated expression compared with normal tissues, being related to poor prognosis, and, moreover, its expression is lower in CAFs compared to normal fibroblasts. These experiments show that the downregulation of FENDRR can activate the PI3K/AKT pathway and enhance the expression of MMP9. Instead, the overexpression of FENDRR had the opposite effect, decreasing MMP9 levels and inhibiting the pro-angiogenic effect of CAFs through the PI3K/AKT pathway, which makes FENDRR a potential therapeutic target for anti-angiogenic therapy for OSCC treatment [74] (Table 3).

Some lncRNAs promote angiogenesis in HNSCC by upregulating VEGF expression, activating pro-angiogenic signaling pathways, sponging anti-angiogenic miRNAs, and modulating the tumor microenvironment. Clinically, lncRNAs, such as MALAT1, HOTAIR, and H19, are associated with increased microvessel density, tumor progression, and poor prognosis.

### 3.4. lncRNAs’ Modulation of Signaling Pathways Involved in HNSCC Invasion and Metastasis

The role of lncRNAs in cancer is not only in mechanisms involved in local tumor growth but also their diverse modes of action, as they potentially participate in various stages of the disease, including invasion and metastasis. The process of cancer metastasis is intricate and involves multiple sequential steps and signaling pathways, which enhance the ability of tumor cells to invade surrounding blood vessels and spread to distant sites within the body. To achieve this, epithelial tumor cells must abandon their phenotype and acquire mesenchymal morphological and transcriptional characteristics, and the process is called epithelial–mesenchymal transition (EMT) [75,76]. In the course of EMT, tumor cells start losing the expression or the function of epithelial markers, such as E-cadherin (epithelial cadherin), involved in intercellular adhesion, and gain mesenchymal markers, such as vimentin, mesenchymal neural cadherin (N-cadherin), matrix metalloproteinases (MMP 1,3,9), integrins (α2β1, α5β1), fibronectin, and collagen [77,78,79]. This transformation is complex and controlled by multiple signaling pathways, including Wnt/β-catenin, RTK (Receptor Tyrosine Kinase), TGF-β, Notch, and Hedgehog [80]. Along these signaling pathways, different transcription factors are involved in the repression of epithelial genes and the activation of the mesenchymal ones, such as Snail family, ZEB (zinc-finger E-box-binding), and TWIST1/2 [81,82].

Recently, it has been confirmed that EMT can be also regulated by lncRNAs, which fulfill their role through regulatory effectors, transcription factors, and signal transduction pathways. During metastasis, functional lncRNAs influence the EMT process by regulating gene expression at different levels, such as chromatin remodeling, transcription, or post-transcription processes [83]. The role of lncRNAs in tumoral processes has been well examined in the recent years, and more and more evidence has emphasized the regulatory potential of lncRNAs in metastasis of HNSCC by either inducing or suppressing EMT, having the capacity to modulate the migration/invasion processes [84].

The most studied lncRNAs in HNSCC promote EMT both in vivo and in vitro. MALAT1 is one of the most studied oncogenic lncRNAs involved in the EMT process. In HNSCC, it is upregulated and correlated with lymph node metastasis and poor prognosis, promoting cell proliferation and invasion in vitro and in vivo. To this end, MALAT1 induces TGF-β and STAT3 overexpression [85] and promotes the activation of Wnt/β-catenin and NF-κB pathways by suppressing VHL, a E3 ligase known to induce the degradation of β-catenin and P65 proteins to inhibit tumor progression [86]. Moreover, in OSCC cells, MALAT1 induces epithelial–mesenchymal transition, being correlated with high levels of vimentin and N-cadherin [87] (Table 4).

HOTAIR is a typical example of carcinogenic lncRNA, which has been found to play a key role in the metastasis of various tumors, including HNSCC. Specifically, HOTAIR has been shown to be highly expressed in OSCC, enhancing the metastatic potential of tumor cells and the expression of EMT markers. In clinical samples, HOTAIR is negatively correlated with the expression of epithelial markers (E-cadherin) and positively correlated with the expression of mesenchymal markers (vimentin, Snail) [88,89] (Table 4).

UCA1 was first discovered in bladder cancer, but it was demonstrated that overexpression of UCA1 also promotes the migration, invasion, and metastasis of other cancer cells, including HNSCC. Here, UCA1 can act as a competing endogenous RNA (ceRNA) sequestering miR-143-3p, indirectly increasing MYO6 expression. In HNSCC, MYO6 expression influences the cytoskeletal rearrangements and focal adhesion dynamics, enhancing the invasive capacity of cancer cells [90]. Thus, aberrant motility enables tumor cells to migrate from their primary site into tissues and lymphatic structures, thereby facilitating local and regional metastasis. UCA1 can also sponge miR-124 to increase TGF-β1 expression, which activates the Notch signaling pathway, promoting EMT and the invasion of TSCC (tongue squamous cell carcinomas) cells [91,92]. Moreover, UCA1 overexpression promoted cell proliferation, invasion, and migration of the LSCC (laryngeal squamous cell carcinoma) cells by activating the Wnt/β-catenin signaling pathway [93]. Several other lncRNAs have been reported to be involved in Wnt/β-catenin cell signaling, being one of the classical pathways that promotes cell proliferation and invasion. lncRNA LINC00473 (Long Intergenic Non-Protein Coding RNA 473) is found to be upregulated in HNSCC cells and to activate the Wnt/b-catenin pathway, inducing invasion and radioresistance [94]. ZFAS1 (ZNFX1 Antisense RNA 1) has a highly upregulated expression in HNSCC cells, correlated with genes involved in multiple pathways, including Wnt/β-catenin, TGF-β, and JAK/STAT [95] (Table 4).

NORAD (Non-Coding RNA Activated by DNA Damage) is a highly conserved lncRNA necessary for genome stability that is dysregulated in various types of cancers, being involved in numerous processes associated with tumor progression. In HNSCC, NORAD interferes with malignant phenotypes and EMT by inhibiting miR-26a-5p, a downstream gene of NORAD. Thus, NORAD knockdown in HNSCC-derived tumor stem cells attenuates migration and invasion, lowers the expression levels of EMT-related markers, like MMP2, MMP9, N-cadherin, and vimentin, and upregulates E-cadherin expression [96].

H19 (imprinted maternally expressed transcript) functions as an oncogene and is overexpressed in HNSCC, being associated with higher invasive capacity of tumor cells and worse overall survival. Increased H19 expression promoted the epithelial–mesenchymal transition, growth, and invasion of ESCC (esophageal squamous cell carcinoma) and NPC by inhibiting E-cadherin expression and modulating the STAT3/EZH2/β-catenin pathway [97].

There are several lncRNAs that act as anti-EMT factors and are negatively correlated with tumor progression. For example, MEG3 (maternally expressed 3) functions as a suppressor in several types of cancers. Ji et al. demonstrated that in HNSCC, the expression of MEG3 was significantly downregulated compared to adjacent normal tissues. Induced overexpression of MEG3 upregulated E-cadherin levels and inhibited cell proliferation and invasion in vitro [98]. NKILA (NF-κB Interacting LncRNA) is another lncRNA demonstrated to have a role in the blockage of tumor growth and the inhibition of metastasis in several types of neoplasms. In cervical squamous cells and carcinoma-derived cells, NKILA suppressed migration and invasion by modulating the EMT processes, as demonstrated by the modified expression level of E-cadherin, N-cadherin, and vimentin [99] (Table 4).

lncRNAs play critical roles in promoting metastasis and invasion in HNSCC by inducing epithelial–mesenchymal transition, remodeling the extracellular matrix, and activating oncogenic signaling pathways, such as PI3K/AKT/mTOR, Wnt/β-catenin, and TGF-β (Figure 2). Clinically, overexpression of lncRNAs, like MALAT1, HOTAIR, H19, and UCA1, is associated with lymph node metastasis, advanced staging, and poor survival, making them promising biomarkers and therapeutic targets for managing aggressive HNSCC.

## 4. Impact of lncRNAs on Therapy Resistance in HNSCC

Despite the progress in treatment methods, various challenges continue to pose significant obstacles for patients. Surgical resection, while often necessary for tumor removal, frequently results in some dysfunction, necessitating numerous corrective procedures. These additional surgeries can lead to substantial physical deformities and profound psychosocial distress, making it difficult for patients to regain a sense of normalcy in their lives. HNSCC is often treated, in addition to surgery, with radiation, chemotherapy (e.g., cisplatin), and targeted therapy (e.g., EGFR inhibitors) [100]. Moreover, both radiation and chemotherapy, although effective in targeting cancer cells, come with a host of severe side effects. Patients frequently experience debilitating toxicities, such as nausea, fatigue, and increased susceptibility to infections, which can drastically diminish their overall quality of life [101,102]. Furthermore, there is a real concern regarding the potential for developing resistance to these treatments, complicating future therapeutic options and potentially resulting in a more aggressive disease course. One of the significant challenges is the risk of locoregional recurrence, which occurs when cancer returns to the original site or nearby areas many years after the initial treatment. This reappearance complicates the clinical management of the disease and also revives the fears and anxieties that patients had hoped to overcome. This situation underscores the long-term psychological burden associated with cancer treatment. Therefore, the major clinical problem is resistance to radiotherapy and chemotherapy, which leads to recurrence and poor survival.

lncRNAs have emerged as key players in these resistance mechanisms [103]. lncRNAs can induce resistance by modulating apoptosis. lncRNAs, such as PVT1, act to inhibit pro-apoptotic signals, which stabilize MYC, inhibit apoptosis, and are responsible for poor chemotherapy response [104]. Another lncRNA, TUG1, upregulates anti-apoptotic proteins (e.g., BCL-2), and it was associated with cisplatin resistance [105]. Radiation and some forms of chemotherapy induce DNA damage, and lncRNAs can boost repair pathways (e.g., homologous recombination). HOTAIR is involved in epigenetic silencing of tumor suppressors and promotes EMT associated with more invasive and resistant cells. These processes were correlated with poor radiotherapy response [106]. Some lncRNAs activating survival signaling can influence the therapy response in HNSCC. H19 is a lncRNA that can act as a molecular sponge for certain miRNAs, such as let-7, which activate Wnt/β-catenin and can induce cisplatin resistance [107]. MALAT1 activates PI3K/AKT, inhibits apoptosis, and can be implicated in chemo- and radioresistance in HNSCC [108] (Table 5).

All of these data show that lncRNAs play a crucial role in therapeutic resistance in HNSCC by promoting anti-apoptotic signaling, enhancing DNA repair, activating survival pathways, such as PI3K/AKT/mTOR, and inducing EMT and cancer stem cell traits (Figure 2).

## 5. Challenges and Future Perspectives

Research into ncRNAs (miRNAs and lncRNAs) is an active field. They have the potential to provide new insights into cell function and develop novel therapeutic strategies for diseases, and they are considered potential therapeutic targets for cancer.

Long non-coding RNAs (lncRNAs) present significant challenges and promising future perspectives in diagnostics. Circulating lncRNAs can be detected in patients’ saliva using state-of-the-art methods, such as qRT-PCR, NGS (Next-Generation Sequencing), and ddPCR (Droplet Digital PCR) [109]. While they hold potential as biomarkers due to their tissue-specific expression and roles in disease processes, they have lower levels than protein-coding genes, making their detection difficult, particularly in body fluids. They have sequence variability and multiple isoforms, making it difficult to design specific and sensitive detection assays. Different lncRNAs have different expression patterns and thus different diagnostic potential.

In this regard, we need to enhance the sensitivity and specificity of lncRNA detection, which will lead to the development of minimally invasive diagnostic and prognostic tools. We can also combine lncRNA expression with other biomarkers or clinical information to improve disease diagnosis, prognosis, and future treatment.

LncRNAs have complex regulatory mechanisms, making them difficult to detect accurately and reliably. There are a few standardized protocols for lncRNA detection, quantification, and data analysis, which restrain reproducibility, which is essential for clinical translation. Indeed, developing and applying SOPs related to lncRNA analysis is imperative for successfully translating lncRNA signatures into clinically meaningful tests. Additionally, establishing reference sample sets for both cancer and standard controls is important for generating a consensus and facilitating validation. Once consensus procedures have been defined and included for profiling lncRNA, it will be possible to interpret and compare different study results and potentially identify lncRNAs acting as specific and sensitive cancer biomarkers. The functions and regulatory roles of many lncRNAs are still being investigated, making it difficult to interpret their expression patterns in disease. Overcoming these challenges through advanced technologies and standardized protocols is crucial for realizing the full potential of lncRNAs in diagnostics.

With a better understanding of the molecular properties of lncRNA, it may be possible to offer a more sensitive and accurate biomarker for early cancer detection or to improve the performance of existing clinical biomarkers.

## 6. Conclusions

This review provides a comprehensive overview that supports the essential role of lncRNAs as key factors in HNSCC carcinogenesis and how they significantly influence signaling pathways involved in regulating cell proliferation, invasion, and survival. We also highlighted the involvement of lncRNAs in treatment resistance, making them important candidates for innovative therapies. Identification of the specific lncRNAs in HNSCC could lead to the development of RNA-based therapies (e.g., lncRNA inhibitors or antisense oligonucleotides) that block their activity, restoring disrupted signaling pathways and sensitizing tumors to chemotherapy and radiotherapy. This information offers new insights for improving clinical outcomes in patients with HNSCC.

It is also important to emphasize that additional studies and well-controlled clinical trials are necessary to fully characterize the promising potential of lncRNAs as diagnostic and prognostic tools and their viability as therapeutic targets in HNSCC.

## Figures and Tables

**Table 1:** Signaling pathways’ modulation by lncRNAs and their effects on HNSCC apoptosis.

Pathway	lncRNAs	Mechanisms	Effect on HNSCC Apoptosis
PI3K/AKT/mTOR	HOTAIR, UCA1, MALAT1	miRNA sponging, PTEN silencing	Inhibit apoptosis
p53	MEG3, HOTAIR, MALAT	Epigenetic silencing and miRNA sponging	Enhance or suppress apoptosis
BCL-2 family	MALAT1, HOTAIR	Epigenetic and ceRNA mechanisms	Block mitochondrial apoptosis
	MEG3, GAS5	Pro-apoptotic signaling	Enhance apoptosis
NF-κB	MALAT1, HNF1A-AS1, HOTAIR	IκB degradation → NF-κB activation	Promote anti-apoptotic genes
JAK/STAT3	LINC00152, MALAT1	↑ STAT3 phosphorylation	Upregulate anti-apoptotic genes
MAPK/ERK	HOTAIR, MALAT, UCA1, AFAP1-AS1	Increase ERK phosphorylation	Anti-apoptotic signaling
	MEG3, GAS5	Inhibit ERK activity	Restore apoptotic sensitivity
Death receptor pathway	HOTAIR, MALAT1	Epigenetic silencing, miRNA sponging	Reduces extrinsic apoptosis

**Table 2:** lncRNAs’ impact on the signaling pathways and their effect on the proliferation process in HNSCC. (↑ - increase).

lncRNA	Signaling Pathway	Effect on HNSCC Proliferation
MALAT1	PI3K/AKT, MAPK, NF-κB	↑ cyclin D1 and CDK4; promotes cell cycle progression and survival
HOTAIR	Wnt/β-catenin	↑ cyclin D1; epigenetically silences p21; enhances proliferation
UCA1	PI3K/AKT	↑ cyclin D1; suppresses p21/p27; increases proliferation
H19	Wnt/β-catenin	↑ cyclin D1; sponges miRNAs (e.g., miR-675); promotes stemness and proliferation

**Table 3:** lncRNAs involved in angiogenesis mechanisms in HNSCC.

lncRNA	Mechanisms	Effect on HNSCC Angiogenesis
HOTAIR	Increases VEGF-A and Ang2	Pro-angiogenic, correlated with advanced stage and poor survival
LINC00319	Increases CCL18, VEGFA, and MMP-9	Enhances proliferation, metastasis, EMT, and angiogenesis
FOXCUT	Increases MMP2, MMP7, MMP9, and VEGF-A	Enhances angiogenesis
FENDRR	Inhibits PI3K/AKT pathway, decreases MMP-9	Anti-angiogenic, inhibits tumor progression

**Table 4:** lncRNAs involved in the modulation of signaling pathways responsible for invasion and metastasis processes in HNSCC.

lncRNA	Mechanism of Invasion/Metastasis	Effect in HNSCC
MALAT1	TGF-β and STAT3 overexpression, activation of Wnt/β-catenin and NF-κB pathways, vimentin and N-cadherin overexpression	Promotes EMT and enhances cell proliferation, invasion, and metastasis
HOTAIR	Downregulates E-cadherin and activates vimentin and Snail expression	Induces EMT, predicts metastasis advanced stage marker
H19	Inhibits E-cadherin, modulates the STAT3/EZH2/β-catenin pathway	Promotes EMT, increases invasion
UCA1	miRNA sponging, Wnt/β-catenin pathway activation	Promotes EMT, migration, and metastasis
LINC00473	Activates the Wnt/b-catenin pathway	Induces invasion and resistance to radiotherapy
ZFAS1	Activates the Wnt/b-catenin, TGF-β, and JAK/STAT pathways	Promotes cell proliferation and invasion
NORAD	Downregulates E-cadherin and upregulates MMP-2, MMP-9, N-cadherin, and vimentin	Promotes EMT, migration, and metastasis
MEG3	Upregulates E-cadherin levels	Inhibits EMT, cell proliferation, and invasion
NKILA	Upregulates E-cadherin and downregulates N-cadherin and vimentin	Inhibits EMT, suppresses migration and invasion

**Table 5:** Key lncRNAs involved in therapy resistance in HNSCC.

lncRNA	Mechanisms	Therapy Resistance in HNSCC
PVT1	MYC, PI3K/AKT activation, apoptosis inhibition	Poor chemotherapy response
TUG1	BCL-2 overexpression	Associated with cisplatin resistance
HOTAIR	Epigenetic silencing of tumor suppressors	Poor radiotherapy response
H19	miRNA sponging, Wnt/β-catenin activation	Induces cisplatin resistance
MALAT1	PI3K/AKT activation, apoptosis inhibition	Poor prognosis, cisplatin resistance, radioresistance

## Data Availability

No new data were created or analyzed in this study.

## Abbreviations

HNSCC	Head and neck squamous cell carcinoma
lncRNAs	Long non-coding RNAs
HPV	Human papillomavirus
HOTAIR	HOX transcript antisense RNA
DNMT1	DNA methyltransferase 1
PVT1	Plasmacytoma variant translocation 1
PRC2	Polycomb repressive complex 2
EZH2	Enhancer of zeste homolog 2
*PTEN*	Phosphatase and TENsin homolog
OSCC	Oral squamous cell carcinoma
*MALAT*	Metastasis-associated lung adenocarcinoma transcript 1
*EMT*	Epithelial–mesenchymal transition
CCAT1	Colon cancer associated transcript 1
*RBPs*	RNA-binding proteins
hnRNPs	Heterogeneous nuclear ribonucleoproteins
SRSFs	Serine/arginine-rich splicing factors
TINCR	TINCR Ubiquitin Domain Containing
ceRNA	Competing endogenous RNA
MEG3	Maternally expressed 3
BCL-2	B-cell lymphoma 2
BCL-XL	B-cell lymphoma-extra large
MYC	Myelocytoma oncogene
BAX	Bcl-2-associated X protein
PUMA	p53 upregulated modulator of apoptosis
NOXA	PMAIP1 (phorbol-12-myristate-13-acetate-induced protein 1)
BAK	Bcl-2 homologous antagonist/killer
XIAP	X-linked inhibitor of apoptosis protein
EGFR	Epidermal growth factor receptor
UCA1	Urothelial cancer-associated 1
PI3K	Phosphatidylinositol 3-kinase
AKT	Serine/threonine kinase 1
GAS5	Growth arrest-specific 5 lncRNA
mTOR	Mammalian target of rapamycin
ASOs	Antisense oligonucleotides
siRNAs	Small interfering RNAs
CRISPR	Clustered regularly interspaced short palindromic repeats
HNF1A-AS1	HNF1A antisense RNA 1 lncRNA
MAPK/ERK	Mitogen-Activated Protein Kinase/Extracellular Signal-Regulated Kinase
IκB	Inhibitor of κB
NF-κB	Nuclear factor kappa-light-chain-enhancer of activated B cells
STAT3	Signal transducer and activator of transcription 3
JAK	Janus kinase
LINC00152	Long intergenic non-protein coding RNA 152
BAD	BCL2-associated agonist of cell death
BIM	Bcl-2 Interacting Mediator of cell death
AFAP1-AS1	Actin filament associated protein 1 antisense RNA 1
FAS	Fas cell surface death receptor
TNF	Tumor necrosis factor
TRAIL	TNF-related apoptosis-inducing ligand
c-FLIP	Cellular FLICE-inhibitory protein
CDKs	Cyclin-dependent kinases
Wnt	Wingless-related integration site
VEGF	Vascular endothelial growth factor
Ang2	Angiopoietin 2
MMP	Matrix metalloproteinases
ECM	Extracellular matrix
NPC	Nasopharyngeal cancer
FOXCUT	FOXC1 upstream transcript
LINC00319	Long intergenic non-protein coding RNA 319
CCL18	Chemokine ligand 18
CAFs	Carcinoma-associated fibroblasts
FENDRR	FOXF1 adjacent non-coding developmental regulatory RNA
TGF-β	Transforming growth factor-beta
MYO6	Myosin VI
VHL	von Hippel–Lindau protein
ZEB	zinc-finger E-box-binding
Snail	Zinc finger protein SNAI1
TWIST1/2	Twist-related protein 1 and 2
TSCC	Tongue squamous cell carcinomas
LSCC	Laryngeal squamous cell carcinoma
LINC00473	Long intergenic non-protein coding RNA 473
ZFAS1	ZNFX1 antisense RNA 1
NORAD	Non-coding RNA activated by DNA damage
ESCC	Esophageal squamous cell carcinoma
NKILA	NF-κB interacting lncRNA
TUG1	Taurine upregulated gene 1
5-FU	5-fluorouracil
